# Antibacterial Coatings for Titanium Implants: Recent Trends and Future Perspectives

**DOI:** 10.3390/antibiotics11121719

**Published:** 2022-11-29

**Authors:** S. Akshaya, Praveen Kumar Rowlo, Amey Dukle, A. Joseph Nathanael

**Affiliations:** 1 Centre for Biomaterials, Cellular and Molecular Theranostics (CBCMT), Vellore Institute of Technology, Vellore 632014, India; 2 School of Advanced Sciences, Vellore Institute of Technology, Vellore 632014, India; 3 School of Bio Sciences & Technology, Vellore Institute of Technology, Vellore 632014, India

**Keywords:** titanium implants, implant-associated infections, antibacterial coatings, 3D printing

## Abstract

Titanium and its alloys are widely used as implant materials for biomedical devices owing to their high mechanical strength, biocompatibility, and corrosion resistance. However, there is a significant rise in implant-associated infections (IAIs) leading to revision surgeries, which are more complicated than the original replacement surgery. To reduce the risk of infections, numerous antibacterial agents, e.g., bioactive compounds, metal ions, nanoparticles, antimicrobial peptides, polymers, etc., have been incorporated on the surface of the titanium implant. Various coating methods and surface modification techniques, e.g., micro-arc oxidation (MAO), layer-by-layer (LbL) assembly, plasma electrolytic oxidation (PEO), anodization, magnetron sputtering, and spin coating, are exploited in the race to create a biocompatible, antibacterial titanium implant surface that can simultaneously promote tissue integration around the implant. The nature and surface morphology of implant coatings play an important role in bacterial inhibition and drug delivery. Surface modification of titanium implants with nanostructured materials, such as titanium nanotubes, enhances bone regeneration. Antimicrobial peptides loaded with antibiotics help to achieve sustained drug release and reduce the risk of antibiotic resistance. Additive manufacturing of patient-specific porous titanium implants will have a clear future direction in the development of antimicrobial titanium implants. In this review, a brief overview of the different types of coatings that are used to prevent implant-associated infections and the applications of 3D printing in the development of antibacterial titanium implants is presented.

## 1. Introduction

**Figure 1:**
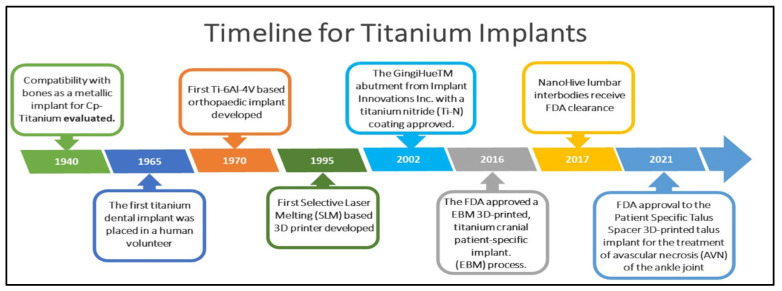
A brief history of the development of titanium implants for biomedical devices.

Implant materials, made up of metals, alloys, ceramics, polymers, and their composites, have been extensively used in the medical field for the past five decades [1]. Pertinently, implants are widely used in orthopedics and dentistry to retain broken bones in an appropriate position or to replace damaged bones [2]. Infection would be the most enduring problem currently facing orthopedic and dental implants, which can cause chronic pain, disability, a lengthy recovery period, and even revision surgery if the implant fails to integrate quickly into the surrounding tissue [3]. An implant must have both strong tissue integration and antimicrobial qualities for it to be effective [2,4]. Most implants fail due to bacterial infections and inadequate tissue integration [5,6]. As a result of their biocompatibility, mechanical characteristics, and corrosion resistance, titanium and its alloys are widely used as implant materials and are considered the best [7]. A brief history of the development of titanium implants for biomedical devices is depicted in Figure 1. However, IAIs can occur due to bacterial infections and the adhesion of bacteria to the surface of titanium implants that, result in the loss of tissue surrounding the implant. IAIs generally occur a few weeks after surgery, but there are also reports of IAIs after several years of implantation [8]. The infection risk of implant materials, which is directly linked to their material state, plays an important role in orthopedic disorders and cannot be ignored.

Moreover, infection of the tissues around implant material is a significant complication of orthopedic surgery [9]. When an infection forms in the area around the implant, not only does the operation and the implant fail, but the patient’s recovery time is also prolonged [3]. Antibiotics are a common solution but have serious side effects that should not be ignored [10]. If the implanted material is infected with bacteria, they form a biofilm, and the positive effects of bactericidal medications could be reduced or eliminated by this biofilm [9,11]. Chemical and physical surface characteristics influence tissue and implant interactions. Most research in recent years has focused more on implant surface design to prevent infections [12]. The implant materials are coated with antibacterial agents via different surface modification techniques to achieve strong osseointegration and anti-infection properties. This review discusses coatings by inorganic antibacterial elements and compounds, antibacterial polymers, peptides, and antibiotics with a major focus on orthopedic and dental applications. Current strategies utilized to create anti-infective titanium implant coatings, multifunctional and smart coatings that facilitate the on-demand release of antibacterial agents, are highlighted. We have also discussed the applications of 3D-printing in the development of antibacterial implants with a real-world 3D printed product which has not been discussed much in other reviews.

## 2. Antibacterial Coatings on Titanium Implants

**Figure 2:**
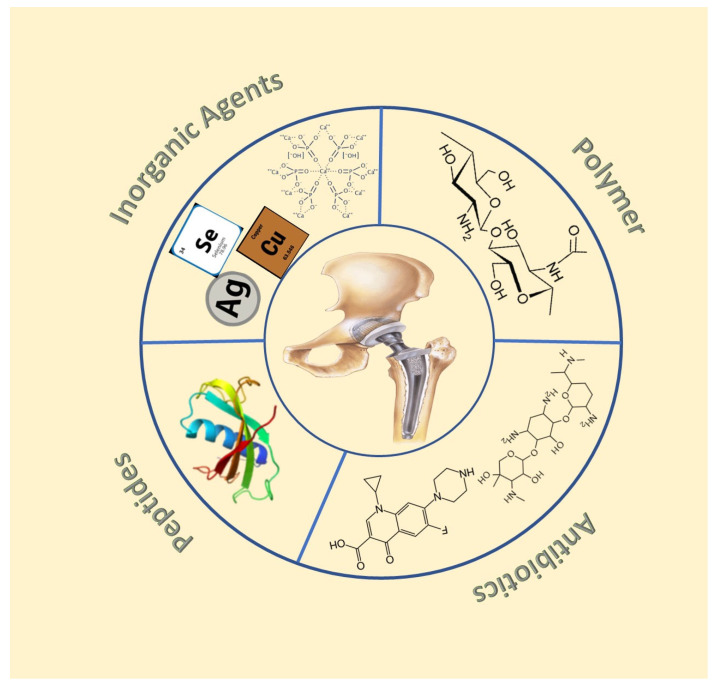
Antibacterial coatings on titanium implants.

Despite the several advantages of titanium alloys as implant materials, they are susceptible to biofilm formation [11], corrosion [13], and even hypersensitivity [14,15]. Coating titanium implants with antimicrobial agents has been one of the common strategies to prevent IAIs [16] (Figure 2). Among the pathogenic bacteria that cause IAIs, the Gram-positive *Staphylococcus aureus* is the most widely studied [17]. *Staphylococcus aureus* can form multicellular biofilms that shield the bacterial cells from the host immune system [18]. Moreover, *Staphylococcus aureus* has evolved to have a variety of defense mechanisms to evade the host immune system, with its biofilms exhibiting enhanced resistance to antibiotics [19]. Other pathogenic bacteria that can cause IAIs include Gram-negative *Pseudomonas aeruginosa*, *Porphyromonas gingivalis*, and *Escherichia coli* and Gram-positive *Staphylococcus epidermidis* and *Streptococcus sanguinis* [20,21,22,23]. Apart from preventing microbial attacks, the coatings should be biocompatible, non-cytotoxic, and promote tissue integration [24]. Coatings generally involve the creation of an additional layer on the surface without disrupting the nature and properties of the bulk material. This can be achieved by various methods, e.g., electrochemical deposition [25], ionized jet deposition (IJD) [26], sol-gel method [27], micro-arc oxidation [28], etc. For the prevention of IAIs, the coatings are loaded with antimicrobial agents, e.g., inorganic elements [29], antibiotics [30], antimicrobial peptides [31], polymers [32], and hybrid inorganic-organic moieties [33], that effectively prevent biofilm formation and enhance tissue integration.

### 2.1. Coatings with Inorganic Antibacterial Agents

To enhance antibacterial activity and create a favorable healing environment, inorganic biofunctional metal ions [34], nanoparticles [35], and non-metals like iodine [36] and fluorine [37] are incorporated into the titanium surface. The incorporation is done either directly or by modifying the titanium surface through various methods, e.g., electrochemical treatment, plasma ion implantation [38], plasma electrolytic oxidation [39], sol-gel [40], micro-arc oxidation [28], etc. The antibacterial nature of these agents is often dictated by the appropriate concentration of the ions and their release into the surrounding tissues [34]. Moreover, as most of the inorganic antibacterial agents are not Gram-specific, there is less chance of antimicrobial resistance compared to antibiotic-loaded coatings [41]. This section deals with coatings containing metal ions, hydroxyapatite (HAP), iodine, carbides, and nitrides.

#### 2.1.1. Metal Doped Coatings

A wide range of metals, particularly transition metals, are doped or co-doped along with other metals onto the titanium surface to enhance the bioactivity of titanium implants [25,42,43,44]. At optimal concentrations, certain metal ions and metal oxide nanoparticles can be therapeutic [33]. The metal ions are often doped into suitable substrates, e.g., TiO_2_ [45,46], bioactive glass [47,48], etc., which are then attached to the titanium implant surface.

##### Silver

By far, silver has been one of the most widely used metals for titanium implant coatings owing to its broad-spectrum bactericidal action [49], biocompatible [50], and stable nature [51]. Studies have also demonstrated the ability of silver nanoparticles to regulate the expression of biofilm–forming genes in *Staphylococcus epidermidis* and *Staphylococcus aureus* [52]. Due to the potential toxicity of high concentrations of silver and silver incorporated systems, care should be taken in controlling their release [53,54]. Silver nanoparticle doped titanium substrates formed through plasma electrolytic oxidation (PEO) of titanium followed by ion implantation produced a strong bactericidal effect against Methicillin-resistant *Staphylococcus aureus* 839, and *Staphylococcus aureus* 224/228, antibiotic-resistant *Escherichia coli K*261, and antibiotic-sensitive *Escherichia coli U*20. The coating’s antibacterial activity and ion release depended on silver ion concentration. An initial fast release of silver ions suitable for preventing infections after implantation was observed, followed by a slow, sustained release for seven days. The ion concentrations were maintained below 300 ppb, which is considered a safe limit for the ion concentration in human blood [55]. In another study, silver enriched porous PEO layer formed using nitrilotriacetic acid (NTA)-based Ag nanoparticles (Ag NP)-loaded calcium–phosphate solution inhibited adhesion and biofilm formation of *Staphylococcus aureus* (strain B 918). The coatings were highly biocompatible, providing an appropriate environment for osteogenic cell growth and proliferation, with increased collagen production around the coated region [56].

##### Copper, Zinc, and Selenium

Next to silver, copper [57], and zinc [58] are the most sought-after dopant metals due to their antibacterial nature, low cost, their ability to promote osteogenesis [51]. Copper and selenium are essential trace elements that the human body needs for proper functioning. Coatings containing these metal ions increase the biocompatibility of the implants [57]. Moreover, it has been reported that zinc-containing coatings help osteoblast differentiation and improve the corrosion resistance of titanium implants [59,60]. Microporous copper-titanium dioxide-coated titanium implants prepared by the micro-oxidation method enhanced implant–bone interface osseointegration when implanted into rabbit femoral condyle.

Furthermore, the coatings were highly biocompatible, promoting the adhesion, proliferation, and differentiation of MC3T3-E1 cells [57]. Titanium substrates containing poly lactic acid (PLA) coatings with different concentrations of CuCl_2_ effectively inhibited the growth of *Staphylococcus aureus* and induced pre-osteogenic effects in vitro and in vivo. In vitro studies showed a dose-dependent burst release of the Cu^2+^ ion and antibacterial action against *Staphylococcus aureus*. The coatings featured cytocompatibility, enhanced facture-end union, and callus formation [61]. A biocompatible zinc-doped hydroxyapatite coating on Ti6Al4V substrate obtained by the solution precursor plasma spraying (SPPS) process exhibited strong antibacterial activity against *Staphylococcus aureus* and moderate activity against *Escherichia coli* [58]. Selenium incorporated onto microporous titanium dioxide coatings with calcium and phosphorus on titanium substrates enhanced the implant’s antibacterial, anti-oncogenic and osteogenic properties. The concentration of calcium and phosphorus was fixed, and that of selenium was varied from 3–14 wt% to assess its bioactivity. The coating loaded with 8 wt% of selenium was found to be optimal, which exhibited 97% eradication of *Escherichia coli* and *Staphylococcus aureus*, maximum osteogenic activity, and anti-oncogenic properties. Higher doses of selenium were found to interfere with cell proliferation, whereas low doses did not cause significant antibacterial action [62].

##### Other Metals

Coatings with other metals, e.g., calcium [63], strontium [64], gallium [65], bismuth [66], and rare earth metals, such as samarium [67], cerium [68], ytterbium, and erbium [69], have also been utilized to enhance the bioactive properties of titanium substrates. Strontium-incorporated ceramics have been reported to enhance bactericidal action and promote bone growth and regeneration [70]. Zinc/Strontium-doped titanium dioxide microporous coating promoted osteoblast adhesion and inhibited the growth of *Staphylococcus aureus* [71]. Titanium substrates coated with bismuth-doped nanohydroxyapatite were bactericidal to *Escherichia coli* and *Staphylococcus aureus* and exhibited improved radiopacity [66]. Dual doping of samarium and strontium on titanium nanotubes significantly enhanced bone integration, biocompatibility, and antibacterial activity [67]. A coating comprising titanium nanotubes doped with gallium demonstrated excellent biocompatibility with osteoblasts and produced significant anti-inflammatory and antibacterial activity [65].

#### 2.1.2. Hydroxyapatite-Based Coatings

Being one of the natural components of bone and teeth, hydroxyapatite (HAP)-(Ca_10_(PO4)_6_(OH)_2_) and nano HAP are one of the most commonly used coating materials for the modification of titanium surfaces [72]. HAPs are well known for their biocompatibility due to their structural and chemical similarity to natural bone and biocidal activity. They can stimulate implant integration by bonding directly to hard tissues [73]. Various bioactive metals, metal oxides, and other elements have been used as dopants to enhance HAP’s antibacterial and biomechanical properties [74]. The hydroxyapatite’s crystal structure permits the small-scale replacement of Ca^2+^ ions with various foreign ions, promoting osteoblast adhesion and enhancing the material’s capabilities as a biomaterial for medical implants [75].

Silver and fluoride incorporated HAP (Ag—FHA) composite coatings, capable of corrosion resistance, were derived from the sol-gel method to prevent bacterial infections on titanium substrate. The antibacterial activity was tested against *Escherichia coli*, and it was dependent on fluoride concentration. The silver ions release was found to be identical in both silver–HAP and silver and fluoride-doped HAP [40]. The contact-killing efficiency of ZnO was utilized in the fabrication of antibacterial coating for titanium substrates. ZnO incorporated HAP eradicated *Escherichia coli* and *Staphylococcus epidermidis* bacteria through reactive oxygen species generation [76]. Niobium-doped hydroxyapatite exhibited excellent biocompatibility, enhanced corrosion resistance, and hemocompatibility. The rare earth metal doped coating showed biocidal nature against *Escherichia coli* and *Bacillus subtilis* [43]. Cerium-incorporated collagen-HAP-based coating formed by a biomimetic method showed high antibacterial activity against *Escherichia coli* and *Staphylococcus aureus* [77]. A plasma-assisted HAP coating with a ternary dopant system comprising 0.25 wt% ZnO, 0.5 wt% SiO_2_, and 2.0 wt% Ag_2_O enhanced bone mineralization. It inhibited the growth of *Escherichia coli* and *Staphylococcus aureus* in vitro 0.65% bone formation was observed with the ternary doped titanium substrate containing the oxides of zinc, silver, and silica. The coating inhibited the growth of *Escherichia coli* and *Staphylococcus aureus,* and the in vivo studies confirmed the non-cytotoxic nature of the coatings [78].

#### 2.1.3. Iodine

Iodine-supported coatings are a good choice for preventing post-operative infections as they have broad-spectrum antibacterial action and some of their compounds also show biocidal effects against viruses and fungi [79,80,81]. Iodine is unlikely to cause adverse effects as it already exists in the body as a part of the thyroid hormone and a trace metal [82]. Povidone-Iodine, commonly known as Betadine, is a well-known iodine-based effective antiseptic used to coat implant surfaces [83]. In a study, iodine-coated titanium implants exhibited antimicrobial activity against Methicillin-sensitive *Staphylococcus aureus* (MSSA), *Pseudomonas aeruginosa*, Methicillin-resistant *Staphylococcus aureus* (MRSA), and Methicillin-Sensitive *Staphylococcus epidermidis* (MSSE) and anti-fungal action against *Candida Albicans*. The antibacterial effectiveness of the iodine-coated titanium surface was higher than that of the anodized titanium surface coating [36]. In another study, calcium titanate-iodine coating formed a novel solution and heat treatment-based ion-exchange reaction that introduced a significant quantity of positively charged iodine ions into titanium, and its alloy surfaces were created. The resultant calcium titanate, which is deficient in calcium, progressively released 5.6 ppm of iodine ions over a period of 90 days. The titanium substrate loaded with 8.6% iodine exhibited antibacterial activity against MRSA, *Staphylococcus aureus*, *Escherichia coli*, and *Staphylococcus epidermidis*, and the antibacterial activity for MRSA persisted for six months when soaked in phosphate buffer solution (PBS) under physiological conditions [84].

#### 2.1.4. Carbides and Nitrides

Inorganic carbides and nitrides are well known for their mechanical strength. These are increasingly utilized in creating strong, robust, antibacterial surfaces for implants that can withstand high corrosion and wear resistance [85]. TiN and SiC coatings deposited on titanium implants capable of preventing peri-implantitis and osteointegration were developed. Both TiN and SiC were quarternized and deposited on the surface of the Titanium substrate to improve the antibacterial activity. The quarternized samples exhibited enhanced antibacterial activity against *Porphyromonas gingivalis* compared to the non-quarternized and non-coated titanium substrates, which can be attributed to the presence of nitrogen atoms that can rupture the cell wall and cell proliferation [86].

### 2.2. Coatings Loaded with Antibiotics

Several antibiotics have been functionalized on titanium surfaces as a coating layer to prevent bacterial adhesion, biofouling adhesion, and biofouling from reducing the risk of infection [87]. Appropriate concentration and optimal drug release are crucial for antibiotic-releasing coating [88]. However, the use of antibiotics on implant surfaces has raised concerns over antibiotic resistance due to the prevalence of antibiotic-resistant bacterial strains [89]. Studies show at least one antibiotic-resistant bacterium in patients with peri-implantitis [90]. Gentamicin is a widely used antibiotic to treat implant-related infections. Belonging to the class of amino glycans, the drug is used to treat a multitude of infections caused by aerobic Gram-negative bacteria, and the efficiency of its bactericidal action depends on its concentration [91]. Gentamicin-based coatings have been reported to enhance osseointegration and prevent osteomyelitis [92], and it is effective in treating infections caused by *Staphylococcus aureus*, *Pseudomonas aeruginosa*, and *Escherichia coli.* Amoxicillin, vancomycin, tetracycline, rifampicin, and levofloxacin are some reported drugs incorporated onto titanium surface to prevent IAIs [93,94,95].

**Figure 3:**
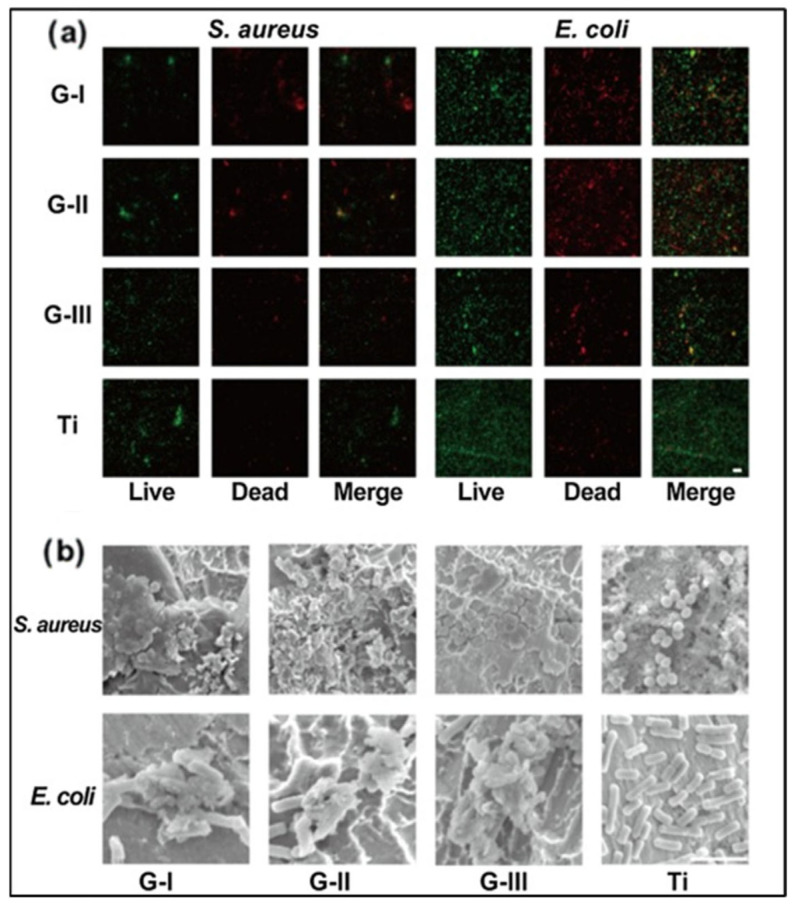
Antibacterial action of graphene-coated Titanium sheets. (**a**) Live/dead fluorescent staining images of bacteria adhered to the graphene-coated Titanium sheets and Titanium sheet. (Live bacteria were stained fluorescent green, and deadly bacteria were stained red.) (Scale bar: 10 μm). (**b**) SEM images showing the bacteria morphology adhered to the surface of the Titanium and graphene-coated sheets. (Scale bar: 10 μm). (Reprinted with permission from [99] Copyright 2021, Elsevier).

Gentamicin-loaded zinc-incorporated halloysites (ZnHNTs)–chitosan was coated onto the titanium foil through electrodeposition. The coatings were cytocompatible and prevented biofilm formation on the titanium, which can be attributed to the release of zinc ions and gentamicin from the coatings. The CS-ZnHNTs-GS coating exhibited large inhibition zones of 3.11 ± 0.79 cm^2^/unit area of the sample, and enhanced cell proliferation was observed in pre-osteoblasts cells [96]. A multilayer coating composed of gentamicin and polyacrylic acid (PAA) (GS/PAA)20/Ti), which exhibited strong antibacterial activity against *Staphylococcus aureus* and *Escherichia coli,* was reported by Li-Jun He et al. [97]. An initial burst release of the drug from the coating was observed for the first 24 h, and a slow, sustained drug release was observed for the next 11 days. This combination of an initial burst release followed by sustained release of gentamicin would be ideal for preventing local infection and bacterial recolonization after implantation. Nearly 100% antibacterial activity against *Escherichia coli was* reported with the drug cefazolin and chitosan coating. The chitosan/cefazolin composites were coated onto the surfaces of Titanium plates using electrodeposition at 10, 30, and 50 V for 3000 s. The Titanium substrates were treated with alkali followed by ultrasonication before electrodeposition. Coatings deposited at 50 V had a more extended drug-release period, greater corrosion resistance in Hank’s balanced salt solution (HBSS), and greater antibacterial activity against *Escherichia coli* than those made at lower potential [98]. Levofloxacin-loaded graphene-coated titanium dental implants effectively prevented bacterial infections after implantation and enhanced biocompatibility [99]. The antibacterial activity of graphene-coated titanium sheets is depicted in (Figure 3). Chitosan, coated with a thin layer of melittin and loaded with the antibiotics vancomycin and oxacillin, exhibited strong bactericidal and anti-biofilm properties. A double layer of chitosan and melittin that had melittin concentrations greater than 0.2 g/mL produced a bactericidal surface that eliminated MRSA bacteria. More than 0.2 g/mL of melittin had a harmful effect on MC3T3 pre-osteoblast cells. Oxacillin (0.16 g/mL) and Melittin (0.05 g/mL) coatings were effective at eliminating MRSA bacteria. In contrast, the coatings containing chitosan/vancomycin (0.16 g/mL)-melittin (0.05 g/mL) destroyed Vancomycin-Resistant *Staphylococcus aureus* (VRSA) bacteria after 6 h. The synergistic effect of melittin and the antibiotics vancomycin and oxacillin caused the disruption of cell wall integrity and was capable of eradicating VRSA and MRSA bacterial strains [95].

### 2.3. Polymer-Based Coatings

Both natural and synthetic polymers are used to create antibacterial surface modifications for titanium implants, as they can be easily functionalized with bioactive moieties. Polymers based on chitosan, nitrogen-containing polyethylene imines, and quaternary ammonium compounds are intrinsically biocidal, while others are incorporated with antibiotics to impart antibacterial activity. Although loading antibiotics onto polymers gives the desired antibacterial effect, they fall short in sustained release, and antibiotics may accumulate in tissues other than the targeted ones. Compared to synthetic polymers, most natural polymers lack mechanical strength and quick degradation that can cause non-uniform elution of the drug particles [100]. Hence, these polymers are often incorporated with inorganic systems, such as metal oxides, HAP, etc., to have efficient antibacterial action. Instead of making the polymer a carrier of antibiotics to have the biocidal effect, bactericidal functionalization can be conducted on the polymeric chain, e.g., attaching a quaternary amine unit that can convert the polymer to have biocidal effects. Chitosan microspheres and nano HAP coatings were loaded with ciprofloxacin into the microspheres using encapsulation and diffusion procedures on titanium surfaces. The coatings were effective against *Staphylococcus aureus*, and the presence of nano HAP and ciprofloxacin levels was found to influence the antibacterial efficiency of the coating [101]. A composite coating comprising poly-L-lysine (PLL)/sodium alginate (SA)/silver nanoparticles capable of preventing bacterial infections and facilitating mineralization in vivo was reported. The release of silver ions from the coating lasted more than 27 days in PBS. Initially, the composite coating exhibited mild cytotoxicity, but when incubated in SBF, it induced mineralization on the surface and exhibited good cytocompatibility [102]. N-halamine-based porous coating (Ti-PAA-NCl) on titanium surfaces capable of providing long-lasting and renewable antibacterial properties was reported for dental implants. Due to the powerful biocidal action against the main pathogenic bacteria of peri-implant infections, the coating can successfully prevent both peri-implant infections in the early stages and post-implant infections before they occur. If peri-implantitis develops after consumption, the active chlorine in N-halamine coating can be regenerated by simple peri-implant irrigation to restore its antibacterial power, which can fight bacteria and biofilm, and therefore considerably enhance the peri-implantitis cure rate [103].

Plasma-polymerized diethyl phosphite (DEP) coated titanium (pp(DEP)-Ti) substrates exhibited excellent cytocompatibility and anti-biofilm properties. The painted surfaces were found to be hydrophobic, and the in vitro studies showed the antibacterial and anticandidal nature of the coating against *Staphylococcus aureus* and *Candida albicans* cells, proving to have a promising potential in treating peri-implantitis [104]. A highly efficient anti-bacterial coating comprising phosphonate/active ester block copolymers (pDEMMP-b-pNHSMA) with identical phosphonate segments was reported. The block copolymer coatings constructed on the surface of the titanium alloy TC4 were prepared by reversible addition-fragmentation chain transfer (RAFT) polymerization. The dense packing of PHMB coating on the titanium alloy surface and the strong coordination between the phosphonate and the titanium substrate enabled nearly 100% eradication of both *Staphylococcus aureus* and *Escherichia coli.* In vivo studies on an infected rat model further confirm the bactericidal nature of the coating [105].

### 2.4. Antimicrobial Peptide-Based Coating

**Figure 4:**
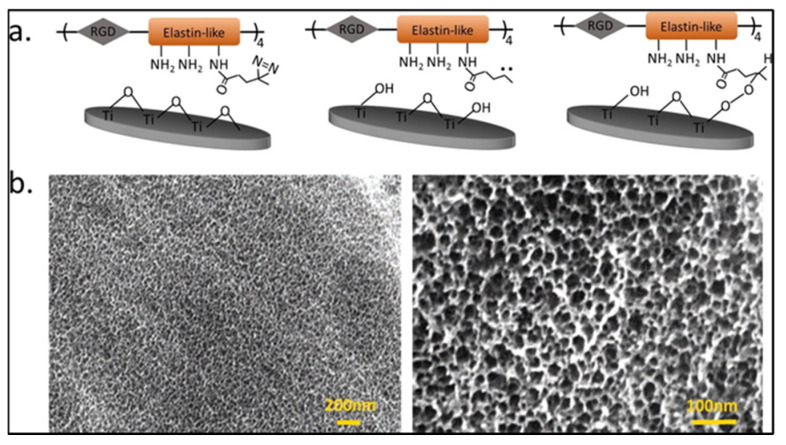
Surface modification of Titanium substrate using elastin-like polypeptide (ELP) (**a**) Shows the schematic model for conjugation of ELP onto Titanium substrates and (**b**) the SEM images of crosslinked ELP onto a Titanium substrate [107] Copyright 2019, Elsevier).

Antimicrobial peptide (AMP) based coatings are now emerging as alternatives to conventional antibiotic-based surface treatments on titanium implants as they exhibit broad spectrum action and require low concentrations for effective microbial resistance [106]. AMPs are usually small having 15–20 amino acids that include both hydrophobic and hydrophilic side chains [31]. They can be cationic or amphipathic and interact with the plasma membrane of bacteria, leading to bacterial cell death [106]. The proline arginine-rich and leucine-rich repeat protein (PRELP) derived antimicrobial peptide RRP9W4N loaded on mesoporous titanium-coated implants exhibited antibiofilm properties equal to that of the drug cloxacillin during the sustained release of the AMP. Osseointegration was unaffected in rabbit tibia models, and a two-fold increase in Bone-to-Implant Contact (BIC%), which refers to the percentage of the implant surface in direct contact with bone, was observed for the AMP-coated samples compared to the control sample [45]. In another study, an antimicrobial coating involving the peptide RRP9W4N, immobilized covalently on titanium, along with elastin-like polypeptide (ELP), containing cell adhesive RGD sequences, exhibited bactericidal action against *Staphylococcus epidermidis*, *Staphylococcus aureus*, and *Pseudomonas aeruginosa* and enabled mammalian osteogenic cell adhesion. Figure 4 shows the surface-modified titanium implant with ELP. Moreover, the AMPs were stable in human blood serum and found to have an antibacterial effect for 24 h [107]. In a different study, multifunctional fluorous-cured collagen coating, loaded with AMPs with Pectolite nanorod (NCS) exhibited antimicrobial activity, biocompatibility, promoted cell adhesion, and at the same time regulated the degradation of the nanorod [108].

### 2.5. Multifunctional and Smart Coatings

Interest in developing multifunctional and smart coatings that have versatile properties, e.g., anti-tumor enhanced corrosion resistance and pH responsiveness, is on the rise. [109] Different strategies have been investigated to overcome antimicrobial resistance and cytotoxic effects of the drugs and prevent early depletion of the drug supply [110]. Targeted and controlled release of drugs from smart coatings can be achieved by responding to different environmental cues like pH and temperature [111]. A smart delivery system for antimicrobial peptides was developed using poly (methacrylic acid) (PMAA), which served as a switch that swelled under normal pH (7.4) and collapsed under a pH less than or equal to 6, indicating a bacterial infection. The titanium substrates were coated with titanium oxide nanotubes (Ti-NTs) by anodic oxidation. They were turned into a box-like structure, onto which the AMPs were loaded and closed with the PMAA pH-responsive gate that opens when there is bacterial infection [112]. An implant coating layer capable of infection-dependent drug release for *Staphylococcus aureus* infections was fabricated using vancomycin and a peptide conjugate with a Staphylococcus sensitive peptide sequence. The tailor-made peptide sequence is linked covalently with the titanium implant surface and releases the antibiotic when a *Staphylococcus aureus* infection arises [113]. In another study, a multi-responsive bacterial anti-adhesion coating that can adapt to the mutable environment was achieved using light-responsive titanium dioxide nanotubes and thermoresponsive P(vinylcaprolactamvinyl caprolactam (VCL)–co-polyethylene glycol methacrylate (PEGMA)–co-alkyl-dimethyl tertiary amine (QAS)–co-vinyl trimethoxysilanetrimethoxy silane (VTMO) copolymer. The generation of ROS species on the titanium composite surface induced light response and reversible conformational change caused the thermo-responsive behavior [114]. A pH-sensitive, self–adaptive coating with antibacterial, anti-inflammatory, and osteointegration was reported by Fanjun Zhang et al. [115]. The smart coating comprised of antibacterial co-polymer containing quaternary ammonium salts (QAs), which were coated on a titanium surface by layer self-assembly. The surface charge reversal of the coatings was confirmed by measuring zeta potential, and the coatings exhibited negative charge in neutral and positive charge in acidic environments. Acidic environments trigger the antibacterial effects of positive control, and this effect is reversed when the pH is high, creating a self-adaptive coating. The coatings were effective against *Escherichia coli* and *Staphylococcus aureus* in vitro. Nano amorphous calcium phosphate (ACP) and titanium dioxide-based coatings showed high corrosion resistance and antibacterial activity. The single-step anodization and anaphoretic deposition were adopted to obtain the coatings. The effect of coatings with and without the addition of chitosan oligosaccharide lactate (ChOL) was observed, and the latter was found to have efficient antimicrobial activity against *Staphylococcus aureus* and *Pseudomonas aeruginosa*. The addition of ChOL increases corrosion resistance by forming a uniform and stable bonding on the cp-titanium surface [116]. Guannan Zhang et al. reported a NIR-II-triggered multifunctional coating containing rare-earth elements, Ytterbium, and erbium-doped onto titanium dioxide nano-shovel/quercetin/L-arginine (Titanium @UCN/Qr/LA) which can simultaneously have anti-bacterial action against *Staphylococcus aureus*, anti-tumor effect against osteosarcoma and promote osseointegration in in vivo and in vitro studies. Adding quercetin suppressed the adverse effects of the dopants—Yb and Er [69]. Titanium implants coated with different coating materials, the composition of the coating, and their antibacterial effects are specified in Table 1.

## 3. Antimicrobial 3D Printed Titanium Implants

The 3D printing of metals allows for the fabrication of customized implants specific to the patient [117]. For the production of titanium implants, selective laser melting (SLM) and electron beam melting (EBM) based 3D printing are most widely used [118]. Although both technologies use a metal powder as a feedstock and a heat source for bonding the metal powder, the surface characteristics broadly vary due to operating conditions. SLM operates under an inert atmosphere, whereas EBM operates under a vacuum, affecting the final product’s overall characteristics [118]. Various strategies, such as coating, surface modification, drug loading, etc., have been employed to achieve antimicrobial capabilities in the implants [119]. Since coatings have already been discussed in the previous sections, the various surface modifications, 3D printing, and design strategies used to develop anti-microbial 3D printed implants are discussed in this section.

The 3D-printed implants are modified using surface modifications to achieve antimicrobial capabilities. Techniques, such as electrochemical anodization or hydrothermal etching, are employed to achieve a unique surface topography of microspheres covered with nanopillars [120]. This topography is found to be effective in the mechanical killing of bacteria. It has also been observed that the surface topography reduces the attachment of bacteria, prevents biofilm formation on the surface of the implant, and inhibits microbial growth [121].

**Figure 5:**
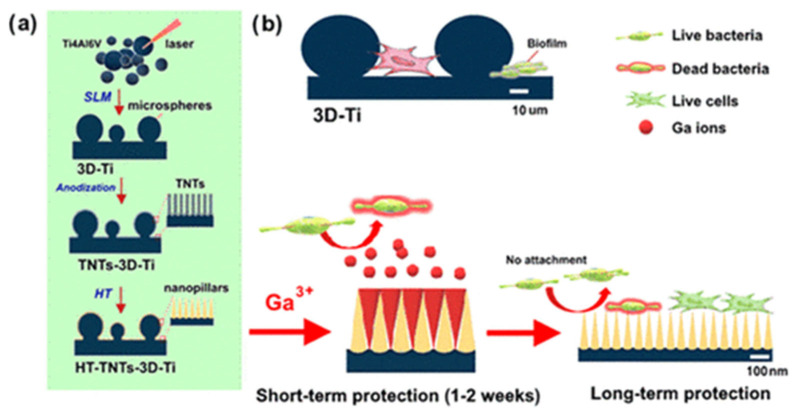
Schematic representation of (**a**)SLM 3D printing and electrochemical anodization process for the fabrication of Titanium implants. (**b**) the mechanism for short and long-term working of the implants [120]. Reprinted with permission from. Copyright 2022 American Chemical Society.

Surface-modified titanium implants loaded with gallium nitrate as the antibacterial agent were studied to achieve antibacterial efficacy from the implants. When tested against *Staphylococcus aureus* and *Pseudomonas aeruginosa*, the local release of gallium ions eradicated bacteria [120]. After the gallium nitrate is released completely, the nanoneedles prevent the attachment of bacteria to the surface. Figure 5 shows the schematic representation of the 3D printing process along with the long- and short-term working of the surface modification and gallium loading on the antibacterial properties.

**Figure 6:**
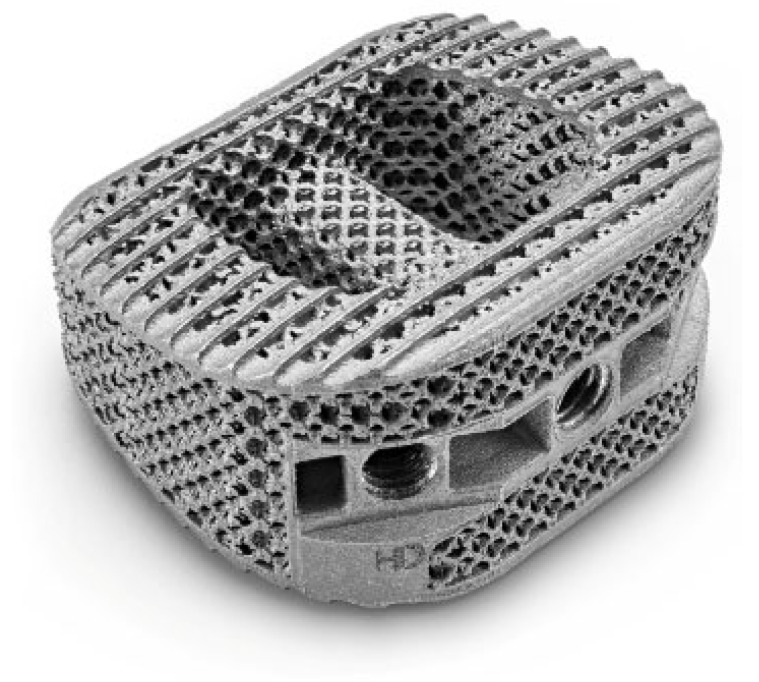
Lumbar interbodies with Hive^TM^ mesh lattice cells. Reprinted with permission from NanoHive (2022) [122].

Besides analyzing the surface characteristics, studies have also attempted to develop implants integrated with novel lattice structures that promote cell adhesion and proliferation. A US-based company, NanoHive medical, has developed Hive^TM^ mesh-based orthopedic implants. These are a novel variety of Ti-6Al-4V implants with a mesh-like structure that promotes osteogenesis and inhibits microbial activity simultaneously [122]. Various HiveTM-based implants have received FDA approval and are being used by surgeons. Figure 6 shows the lumbar interbodies with HiveTM-based lattice structure.

Another strategy employed for achieving the antimicrobial effect is the loading of antibiotic drugs. These drugs are released at the target site, inhibiting bacterial growth. Titanium implants are designed to comprise local reservoirs that can release antibiotic drugs. Titanium implants with specially designed reservoir and polylactic acid (PLA) implant loaded with doxycycline were compared for their anti-microbial activity. It was found that, due to the design considerations of the powder-based 3D printing process, the titanium implants showed better antibacterial capabilities [123].

Although drug-loaded titanium implants have shown satisfactory antibacterial capabilities, they have a long way to go until they are considered the ‘gold standard’. One study compared the antimicrobial activity of tantalum, 3D printed porous titanium, and smooth titanium alloy with antibiotic-loaded bone cement against methicillin-resistant and sensitive *Staphylococcus aureus*. The metal disks were loaded with vancomycin and studied for their microbial inhibition capabilities using agar plates. It was observed that for Methicillin-sensitive *Staphylococcus aureus*, smooth titanium alloys showed microbial inhibition for a short duration of two days. The 3D porous titanium beads also showed better inhibition compared to antibiotic-loaded cement and tantalum over a period of seven days. For Methicillin-sensitive *Staphylococcus aureus*, tantalum and 3D porous titanium showed a larger zone of inhibition than antibiotic-loaded cement for three days. However, antibiotic-loaded cement showed a larger zone of inhibition compared to the vancomycin-loaded tantalum and porous titanium beads over a period of seven days [124].

## 4. Conclusions

Many researchers have already focused on improving titanium implants through antibacterial coatings to alter the implant surface. This review aims to provide an overview of different types of antibacterial coatings that help impart antibacterial nature and improve the overall function and lifetime of titanium implants. Silver continues to be the most explored metal for antibacterial coating among metals. As many studies have confirmed the anti-oncogenic effects of selenium, the metal is sought for multifunctional coatings required to provide the anti-oncogenic effect. The surface topography of the titanium implant plays an important role in delivering the desired antibacterial effects. For example, TiO_2_ nanotubes and porous titanium surfaces serve as reservoirs for antibacterial agents and tailor their local release. The correct use of click chemistry reactions for surface modification ensures robust antibacterial peptides and polymeric coating attachment onto the implant surface. The convergence of different fabrication techniques, e.g., phototherapy, anodic oxidation, micro-arc oxidation, and electrophoretic deposition, paves the way for smart and multifunctional coatings. However, a major drawback in the construction of multifunctional coatings is that the use of different techniques increases the complexity and construction time of the implant. Furthermore, huge investment is needed for equipment used for techniques such as sputtering. Moreover, due to the complexity of the coating technologies, it is challenging to meet the requirements established by national legislators and regulatory organizations, which hinders the commercial feasibility of multifunctional coatings.

## 5. Future Perspectives

Multifunctional and stimuli-responsive coatings will have a prominent role in the near future. However, multifunctionality cannot be achieved with a single anti-bacterial agent. A combination of surface modification technologies and antibacterial agents is essential to construct an ideal implant surface capable of inhibiting bacterial adhesion, promoting osseointegration and osteoinduction. Using such antibacterial agents in combination should not induce cytotoxicity, as bio-active metals like silver can induce cytotoxicity if the right concentration is not incorporated. While trying to impart multifunctional properties, often, two or more technologies are utilized, increasing the cost and duration of implant construction. Developing coatings with controlled/sustained drug release remains a challenge for researchers, and more research should be focused on modifying the implant surface that can achieve sustained drug release and can also prevent antimicrobial resistance. Renewable antibacterial coatings, whose antibacterial action can be replenished by simple methods, e.g., rechlorination with sodium hypochlorite, hold a promising potential in preventing peri-implantitis. Studies with cyclic antibacterial tests would be more reliable for clinical translation [93]. To address the limitations in the use of antibiotics in implant coatings, smart coatings that release drug molecules in response to enzymes released by the host tissues or to pH changes are being explored widely. These stimuli-responsive drug release strategies can help achieve sustained drug release and reduce the risk of antibiotic resistance. The use of quorum-sensing inhibitors and immunomodulators in implant coatings is in its early stages and has a prominent role in the construction of antibacterial surfaces for biomedical devices [125,126]. Implant materials are considered foreign substances by the immune system, and conventional methods usually neglect the effect of immune response and focus majorly on optimizing the osteogenic properties of the implant. Osteo-immunomodulation has opened a new pathway for antibiotic-free coatings, which can create a local environment that retards bacterial colonization [125]. Such coatings need to be carefully designed as they directly interfere with the innate immune system. To overcome the long duration, cost, and complexity of implant coatings, an intraoperative coating that can be prepared in less than ten minutes was reported for the first time by Weixian Xi et al. [95]. The coating exhibited in vivo efficiency against *Staphylococcus aureus* in two weeks post-arthroplasty and post-spinal surgery infections. Intra-operative coatings that require less preparation time and provide long-term protection against bacterial infections can play a major part in the development of antibacterial dental implants. The development of customized patient-specific implants has increased with the advent of additive manufacturing into biomedical devices. While 3D printed titanium implants present an efficient as well as convenient choice compared to the implants manufactured by conventional processes, the cost and scalability of 3D printed titanium implants are two crucial factors that need to be considered, and more research needs to be done on the development of biocompatible alloys for the 3D printing of titanium implants.

## Figures and Tables

**Table 1:** Different types of titanium implant coatings and their antibacterial effects.

Coating Type	Composition	Major Technique	Antibacterial Efficiency (In Vitro Studies)	Reference
Inorganic ions/elements	Ag-doped TiO_2_	Plasma Electrolytic Oxidation (PEO)	Significant reduction (*p* < 0.05) in cell numbers of *Escherichia coli* and *Staphylococcus aureus* and metabolic activity.	[55]
			High silver content samples showed a 6-log reduction of *Staphylococcus aureus*	
	Ag nanoparticles (Ag NP)-loaded calcium–phosphate solution	Plasma Electrolytic Oxidation	Antibacterial action aginst *Staphylococcus aureus* (strain B 918)	[56]
	Zn doped HAP	Solution Precursor Plasma Spray (SPPS) process	Antibacterial action aginst *Escherichia coli* and *Staphylococcus aureus*	[58]
	Selenium incorporated onto microporous titanium dioxide coatings with Ca and P on titanium substrates	Micro-arc oxidation (MAO)	A minimum of 94% killing activity after 28 days was observed for *Escherichia coli* and *Staphylococcus aureus* in in vitro studies	[62]
	Zn/Sr-doped titanium dioxide	Micro-arc oxidation	Fewer surviving colonies of *Staphylococcus aureus* was observed in spread plate analysis	[71]
	Bismuth doped nanohydroxyapatite	Alkali-thermal treatment	Large zone of inhibiton was observed for *Escherichia coli* and *Staphylococcus aureus*	[66]
	Samarium and Strontium on TiO_2_ nanotubes	Anodization	Nearly 100% antibacterial activity was observed for *Escherichia coli* and *Staphylococcus aureus* in plate counting experiments	[67]
			Zone of inhibition was larger for *Staphylococcus aureus* than *Escherichia coli.*	
	TiO_2_ nanotubes doped with gallium	Anodization and solvent casting	Low concentration of live Staphylococcus aureus and *Escherichia coli* was observed in Live/Dead cell assay	[65]
	Ag–HAP-fluoride	Sol-gel	96% reduction in *Escherichia coli* was observed after 6 h in spread plate results for Ag—HAP-fluoride coating which had 0.3% *w*/*v* of Ag and P/F ratio of 6	[40]
	ZnO-HAP	Spin coating	Drastic reduction in the colonies of *Escherichia coli* and *Staphylococcus aureus* was observed after 4 h of incubation	[76]
	Niobium doped hydroxyapatite	Microwave irradiation	Large zone of inhibition was observed for *Escherichia coli* and *Bacillus subtilis*	[43]
	Cerium incorporated collagen-HAP	Immersion of the titanium substrate in supersaturated calcification solution (Ce-SCS)	After 24 h incubation 92.61% *Escherichia coli* and 73.59% *Staphylococcus aureus* were eradicated	[77]
	Calcium Titanate-Iodine coating	Solution and heat treatment method that controllably incorporates 0.7% to 10.5% of Iodine into Titanium	About 99% of bactericidal activity was observed for *Methicillin-resistant Staphylococcus aureus*, *Staphylococcus aureus*, *Escherichia coli*, and *Staphylococcus epidermidis*	[84]
	TiN and SiC coating	Magnetron sputtering, Plasma-enhanced chemical vapor deposition system (PECVD)	Reduction in number of live *Porphyromonas gingivalis* was observed after 4 h of incubation.	[86]
Antibiotic based	Gentamicin loaded zinc–incorporated Halloysites (ZnHNTs)–Chitosan	Electrodeposition (EPD)	Inhibition zones of 3.11 ± 0.79 cm^2^/unit area of the sample was observed in disc diffusion assay for *Staphylococcus aureus*	[96]
	Gentamicin and polyacrylic acid (PAA)	Layer-by-layer assembly	About 99% of bactericidal activity was observed for *Staphylococcus aureus* and *Escherichia coli*	[97]
	Chitosan/cefazolin	Electrophoretic deposition	Nearly 100% bactericidal activity against *Escherichia coli* was shown by the coating which has the highest drug concentration	[98]
	Levofloxacin loaded graphene coating	Sandblasting, large-grit, acid-etching and salinization	Large bacteriostatic circle diameters were observed for Staphylococcus aureus and *Escherichia coli*	[99]
	Chitosan, coated with a thin layer of melittin and loaded with the antibiotics vancomycin and Oxacillin	Spin coating and casting method	The coatings were able to eradicate *Methicillin-resistant Staphylococcus aureus* (MRSA) and *Vancomycin Resistant Staphylococcus aureus* (VRSA) at the early stages	[95]
	poly-L-lysine (PLL)/sodium alginate (SA)/Silver nanoparticles	Electrostatic self-assembly, dip coating	A distinct ring was observed in zone of inhibition test for *Staphylococcus aureus* and *Staphylococcus mutans*	[102]
	N-halamine based porous coating (Ti-PAA-NCl)	Alkali-heat treatment, surface grafting and N-Cl functionalization	Bactericidal rate of 96% for *Staphylococcus aureus* and 91% *Porphyromonas gingivalis* was observed in contact killing assay	[103]
	Diethyl phosphite (DEP) coated Titanium (_pp_ (DEP)-Ti)	Plasma polymerization	The number of *Staphylococcus aureus* and *Candida albicans* colonies decreased after 24 h	[104]
	Phosphonate/active ester block copolymers (pDEMMP-b-pNHSMA) and PHMB	Reversible addition-fragmentation chain transfer (RAFT) polymerization.	Nearly 100% antibacterial activity was observed for *Staphylococcus aureus* and *Escherichia coli*	[105]
Antimicrobial Peptide based	A recombinant elastin like peptide coating with cell- adhesive RGD sequences with a covalently attached AMP, RRP9W4	Covalent immobilization of AMPs to titanium surface	The number dead *Staphylococcus epidermidis*, *Staphylococcus aureus,* and *Pseudomonas aeruginosa* cells increased after 48 h	[107]
	RRP9W4N incorporated into mesoporous TiO_2_.	Spin-coating	Bactericidal action against *Staphylococcus epidermidis*	[45]
	Pectolite nanorod (NCS) with AMP-loaded collagen shell	Microarc oxidation (MAO), Spin coating	Contact killing efficiency was almost 100% for *Staphylococcus aureus*	[108]
Multifunctional Coatings	poly (quaternary ammonium salts-co-methacrylic acid) (PQA)	Anodic Oxidation and Spray coating	Efficient antibacterial action was observed against *Escherichia coli* and *Staphylococcus aureus*	[114]
	P (vinylcaprolactam (VCL)–co-polyethylene glycol methacrylate (PEGMA)–co-alkyl-dimethyl tertiary amine (QAS)–co-vinyltrimethoxysilane (VTMO)) copolymer/ TiO_2_ nanotube	Layer-by-layer (LbL) self-assembly method	Antibacterial action was observed at lower pH for *Staphylococcus aureus* and *Escherichia coli*	[115]
	Nano amorphous calcium phosphate (ACP) and titanium dioxide with chitosan oligosaccharide lactate (ChOL)	Anodization and anaphoretic electrodeposition	Three-to-four-fold reduction in the number of *Staphylococcus aureus* and *Pseudomonas aeruginosa* colonies was observed after 420 min	[116]
	poly (methacrylic acid) (PMAA) loaded onto TiO_2_ nanotubes (Ti-NTs) with HHC36 peptides, with a sequence of KRWWKWWRR	Anodization	99% of bactericidal activity was observed for Staphylococcus aureus, *Escherichia coli*, *Pseudomonas aeruginosa*, *Methicillin-resistant Staphylococcus aureus*	[112]
	Yb and Er doped Ti nano -shovel/quercetin/L-arginine (TiO_2_@UCN/Qr/LA)	Phototherapy	Above 90% bactericidal action against *Staphylococcus aureus*	[69]

## Data Availability

Not applicable.
